# A European Renal Association (ERA) synopsis for nephrology practice of the 2023 European Society of Hypertension (ESH) Guidelines for the Management of Arterial Hypertension

**DOI:** 10.1093/ndt/gfae041

**Published:** 2024-02-14

**Authors:** Pantelis Sarafidis, Roland Schmieder, Michel Burnier, Alexandre Persu, Andrzej Januszewicz, Jean-Michel Halimi, Mustafa Arici, Alberto Ortiz, Christoph Wanner, Giuseppe Mancia, Reinhold Kreutz

**Affiliations:** 1st Department of Nephrology, Aristotle University of Thessaloniki, Hippokration Hospital, Thessaloniki, Greece; Department of Nephrology and Hypertension, University Hospital Erlangen, Germany; Faculty of Biology and Medicine, University of Lausanne, Lausanne, Switzerland; Division of Cardiology, Cliniques Universitaires Saint-Luc and Pole of Cardiovascular Research, Institut de Recherche Expérimentale et Clinique, Université Catholique de Louvain, Brussels, Belgium; Department of Hypertension, National Institute of Cardiology, Warsaw, Poland; Service de Néphrologie-Hypertension, Dialyses, Transplantation rénale, CHRU Tours, Tours, France and INSERM SPHERE U1246, Université Tours, Université de Nantes, Tours, France; Department of Nephrology, Hacettepe University Faculty of Medicine, Ankara, Turkey; Department of Nephrology and Hypertension, IIS-Fundacion Jimenez Diaz UAM, Madrid, Spain; Division of Nephrology, University Hospital Würzburg, Germany; University of Milano-Bicocca, Milan, Italy; Charité-Universitätsmedizin Berlin, Corporate Member of Freie Universität Berlin, Humboldt-Universität zu Berlin, and Berlin Institute of Health, Institut für Klinische Pharmakologie und Toxikologie, Berlin, Germany

**Keywords:** albuminuria, blood pressure, chronic kidney disease, hypertension, hypertensive kidney disease

## Abstract

In June 2023, the European Society of Hypertension (ESH) presented and published the new 2023 ESH Guidelines for the Management of Arterial Hypertension, a document that was endorsed by the European Renal Association (ERA). Following the evolution of evidence in recent years, several novel recommendations relevant to the management of hypertension in patients with chronic kidney disease (CKD) appeared in these Guidelines. These include recommendations for target office blood pressure (BP) <130/80 mmHg in most and against target office BP <120/70 mmHg in all patients with CKD; recommendations for use of spironolactone or chlorthalidone for patients with resistant hypertension with estimated glomerular filtration rate (eGFR) higher or lower than 30 mL/min/1.73 m^2^, respectively; use of a sodium-glucose cotransporter 2 inhibitor for patients with CKD and estimated eGFR ≥20 mL/min/1.73 m^2^; use of finerenone for patients with CKD, type 2 diabetes mellitus, albuminuria, eGFR ≥25 mL/min/1.73 m^2^ and serum potassium <5.0 mmol/L; and revascularization in patients with atherosclerotic renovascular disease and secondary hypertension or high-risk phenotypes if stenosis ≥70% is present. The present report is a synopsis of sections of the ESH Guidelines that are relevant to the daily clinical practice of nephrologists, prepared by experts from ESH and ERA. The sections summarized are those referring to the role of CKD in hypertension staging and cardiovascular risk stratification, the evaluation of hypertension-mediated kidney damage and the overall management of hypertension in patients with CKD.

## INTRODUCTION

In June 2023, the European Society of Hypertension (ESH) presented and published the new 2023 ESH Guidelines for the Management of Arterial Hypertension [1], a document that was endorsed by the European Renal Association (ERA). Following recent evidence, several novel recommendations relevant to the management of hypertension in patients with chronic kidney disease (CKD) appeared in these Guidelines. The present report is a synopsis of the sections of the ESH Guidelines that are relevant to the daily clinical practice of nephrologists, prepared by experts from ESH and ERA. The sections summarized are those referring to the role of CKD in hypertension staging and cardiovascular (CV) risk stratification, the evaluation of hypertension-mediated kidney damage and, most importantly, the management of hypertension in patients with CKD. Of note, the 2023 ESH Guidelines and the present document do not discuss issues relevant to hypertension in patients with CKD G5 on chronic dialysis treatment; this field is extensively discussed in a previous joint consensus statement of the two societies [2, 3].

## DEFINITION OF HYPERTENSION AND CLASSIFICATION OF BP

In the 2023 ESH Guidelines, hypertension is defined based on repeated office systolic blood pressure (SBP) values ≥140 mmHg and/or diastolic BP (DBP) ≥90 mmHg [1]. The document acknowledges that this definition is arbitrary and has mainly the pragmatic purpose of simplifying the diagnosis and decision on hypertension management, as there is a continuous relationship between BP and the risk of death from stroke or ischemic heart disease starting from an office SBP >115 mmHg and a DBP >75 mmHg [4]. In this context, the above office threshold BP values correspond to the level of BP at which the benefits of intervention (lifestyle interventions or drug treatment) exceed those of inaction, as shown by outcome-based randomized controlled trials (RCTs). The classification of office BP and definition of hypertension grades remain the same from previous guidelines and are presented in Table 1. The evidence grading system used in the 2023 ESH Guidelines is depicted in Supplementary data, Fig. S1.

**Table 1:** Classification of office BP and definition of hypertension grades in adults and adolescents ≥16 years old (from [1], with permission).

**Category**	**Systolic and diastolic blood pressure (SBP and DBP)**
Optimal	SBP <120 and DBP <80 mmHg
Normal	SBP 120–129 and DBP 80–84 mmHg
High-normal	SBP 130–139 and/or DBP 85–89 mmHg
Grade 1 hypertension	SBP 140–159 and/or DBP 90–99 mmHg
Grade 2 hypertension	SBP 160–179 and/or DBP 100–109 mmHg
Grade 3 hypertension	SBP ≥180 and/or DBP ≥110 mmHg
Isolated systolic hypertension^a^	SBP ≥140 and DBP <90 mmHg
Isolated diastolic hypertension^a^	SBP <140 and DBP ≥90 mmHg

The BP category is defined by the highest level of BP, whether systolic or diastolic.

a Isolated systolic or diastolic hypertension is graded 1, 2 or 3 according to SBP and DBP values in the ranges indicated.

In addition to grades of hypertension, which are based on BP values, the Guidelines also distinguish stages of hypertension as summarized below [Class of Recommendation (CoR) I, Level of evidence (LoE) C] [1]. The presence of CKD plays a crucial role in this staging.

• Stage 1: uncomplicated hypertension [i.e. without hypertension-mediated organ damage (HMOD), established CV disease (CVD) and CKD G3 or higher].
• Stage 2: presence of HMOD or CKD G3 or diabetes.
• Stage 3: established CVD, or CKD G4 or G5.

## RECOMMENDATIONS RELEVANT TO THE DIAGNOSIS OF HYPERTENSION-MEDIATED KIDNEY DAMAGE AND OTHER DIAGNOSTIC CONSIDERATIONS

The 2023 ESH Guidelines list assessment of serum creatinine (SCr), estimation of glomerular filtration rate (eGFR) with the 2009 CKD-Epidemiology Collaboration formula [5] and evaluation of urine albumin:creatinine ratio (ACR) measured from a spot urine sample (preferably early morning urine) as two of the three (the third being 12-lead electrocardiogram) basic tests to assess HMOD and stage hypertension. These examinations should be documented in all patients upon hypertension diagnosis, and at least annually thereafter [1]. Serum creatinine alone is identified as an insensitive marker of renal impairment, because a major reduction in kidney function can occur before SCr rises. It is also suggested that a negative urinary dipstick test does not rule out A2 albuminuria, as many times it cannot detect ACR levels at the lower range [6], but it can offer information on other signs of kidney injury (i.e. microscopic hematuria, active urine sediment) and should be performed at least at the initial evaluation. The document endorses the currently universally used definition for CKD, involving an eGFR <60 mL/min/1.73 m^2^ at any level of albuminuria or an ACR >30 mg/g at any levels of eGFR persisting for more than 3 months, and the current nomenclature for albuminuria, to highlight the risk associated to albuminuria increase, i.e. (i) normal/mildly increased, ACR <30 mg/g (A1, formerly termed normoalbuminuria); (ii) moderately increased, ACR 30–300 mg/g (A2, formerly termed microalbuminuria); and (iii) severely increased, ACR >300 mg/g (A3, formerly termed macroalbuminuria) [7].

Kidney ultrasound is listed among the extensive examinations for HMOD, due to its low cost, widespread availability and useful information on renal morphology (kidney size and structure, roughness, adiposity, kidney stones) [8]. The role of spectral Doppler ultrasound with evaluation of renal resistive index (RRI), a reproducible measure of renal arterial impedance, as initial screening for renal artery stenosis is also emphasized. A RRI value lower than 0.7 is traditionally indicating normal impedance to renal blood flow, although considerable heterogeneity has been reported [9].

The clinical indications for performance of home BP monitoring (HBPM) and ambulatory BP monitoring (ABPM) are not largely different between hypertensive patients with or without CKD. However, the guidelines highlight the increased prevalence of masked hypertension and high night-time BP with abnormal dipping status in patients with CKD [10–13] as specific indications for HBPM and ABPM, respectively, in these individuals (CoR I, LoE B) [1].

## THE POSITION OF CKD IN ASSESSING THE OVERALL CARDIOVASCULAR RISK IN PATIENTS WITH HYPERTENSION

**Figure 1:**
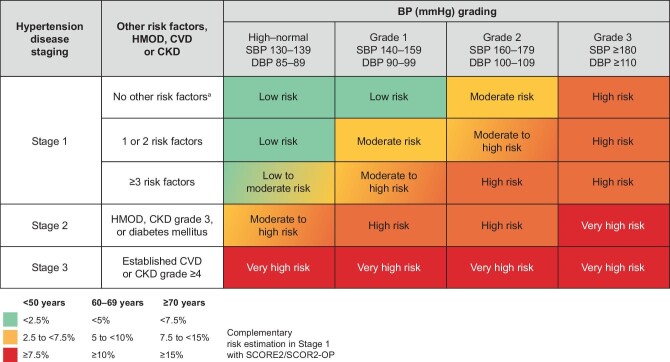
CV risk stratification according to grade and stage of hypertension (from [1], with permission). HMOD: hypertension-mediated organ damage, defined as increased large artery stiffness, non-hemodynamically significant atheromatous plaque (stenosis) on imaging, left ventricular hypertrophy, CKD G1–G2/A2 (i.e. albuminuria 30–300 mg/g with eGFR ≥60 mL/min/1.73 m^2^) or G3 (i.e. eGFR 30–59 mL/min/1.73 m^2^), ankle–brachial index <0.9 or advanced retinopathy.

Among several factors that influence CV risk in patients with hypertension, the 2023 ESH Guidelines promptly identify a lower eGFR and a higher albuminuria, indicating loss of kidney function and kidney damage, respectively, as independent and additive predictors of increased CV risk, in addition to being risk factors for progression of kidney disease [14, 15]. CKD A2 (moderately increased albuminuria, ACR 30–300 mg/g) or CKD G3 (eGFR 30–59 mL/min/1.73 m^2^) are listed as features identifying HMOD, while CKD A3 (severely increased albuminuria, ACR >300 mg/g) and CKD G4 and G5 (eGFR <30 mL/min/1.73 m^2^) are listed among features identifying established kidney disease [1]. As such, the presence of CKD is exemplified as a main factor in the proposed system for overall CV risk stratification in patients with hypertension (Fig. 1).

## TREATMENT OF HYPERTENSION IN CKD

### Initiation of treatment

The 2023 ESH Guidelines recommend that in patients aged 18–79 years, the office threshold for initiation of drug treatment is 140 mmHg for SBP and/or 90 mmHg for DBP (CoR I, LoE A) [1]. The exception to this rule is adult patients with a history of CVD, predominantly coronary artery disease, in whom drug treatment should be initiated in the high-normal BP range (SBP ≥130 or DBP ≥80 mmHg) (CoR I, LoE A). Nephrologists should note that many patients with CKD are falling in the latter category. In patients ≥80 years old, the recommended office SBP threshold for initiation of drug treatment is 160 mmHg (CoR I, LoE A), but a lower SBP threshold of 140–159 mmHg may be considered (CoR II, LoE B).

### Treatment targets

The 2023 ESH Guidelines offer a detailed discussion on the issue of the best (most protective) BP targets in patients with CKD, including those with CKD and diabetes mellitus (DM), recognizing that for more than a decade, there has been considerable debate in the scientific literature in this field [1]. Old observational data suggested an association between BP and the risk for kidney failure, starting from a SBP level of >120 mmHg [16]. More recent data from China obtained in CKD patients without antihypertensive therapy followed prospectively for 5 years indicated that a BP >130/90 mmHg was associated with a significantly increased risk of CV and kidney outcomes [17]. However, RCT evidence to fully delineate the target BP in CKD is missing; ideally, this would be an RCT comparing different BP targets (i.e. SBP <140 vs <130 mmHg), including a CKD population with an appropriate mixture of kidney function levels, albuminuria levels and etiology, achieving corresponding BP levels during follow-up and being powered to investigate CV outcomes, hard kidney outcomes and mortality.

Current evidence in the field comes mainly from two previous trials in non-diabetic CKD that randomized patients to different levels or ranges of mean BP and examined kidney outcomes. In the Modification of Diet in Renal Disease (MDRD) study the projected GFR decline within 3 years, and the risk of end-stage kidney disease (ESKD) and death were not significantly different between groups of low and usual BP target [18]. However, analyses by baseline proteinuria showed that those with proteinuria >1 g/day in the low-target group had a decrease in protein excretion and a slower GFR decline over time compared with patients in the usual-target group [19]. Similarly, in the African American Study on Kidney Disease (AASK) no difference in outcomes between BP target groups was observed in the overall population [20]; in a *post hoc* analysis, again, low BP was associated with better kidney outcomes in the small subset of patients with proteinuria >1 g/day [21]. Subsequent analyses of MDRD and AASK combined the randomized trial periods with subsequent observational follow-up phases. In MDRD long-term analysis, low-target BP was associated with overall reduced risk for ESKD and the composite of ESKD or death, but this was again mainly driven by a beneficial effect in patients with baseline proteinuria >1 g/day [22]. In the AASK long-term follow-up analysis, no difference in the risk of the composite outcome of doubling of SCr, ESKD or death was noted. However, for patients with urine protein-to-creatinine ratio (PCR) >0.22 g/g, which roughly equals proteinuria of 0.25–0.3 g/day and urine ACR >100 mg/g in most patients, there was a beneficial effect with low BP [23]. A subsequent analysis combining the trial and cohort periods of both these trials (adding up to 1907 patients and a median follow-up of 14.9 years), showed that low target BP was associated with significant reductions in the risks of ESKD and mortality in the total population; and this effect was mainly driven by changes in patients with urine PCR >0.44 g/g (urine ACR roughly >200 mg/g) [24]. Thus, sustainability of BP reduction and extent of proteinuria are major determinants of nephroprotection in patients with non-diabetic CKD.

The 2023 ESH Guidelines suggest that the results of the Systolic Blood Pressure Intervention Trial (SPRINT) have little relevance to the question discussed herein [1]. SPRINT randomized 9361 hypertensive patients of increased CV burden to intensive (target SBP <120 mmHg) or standard (target SBP <140 mmHg) treatment [25]. Of these patients, about 28% had CKD with eGFR between 20 and 60 mL/min/1.73 m^2^, but very few had albuminuria A2 or A3, as individuals with proteinuria >1 g/day or >1 g/g were excluded. Importantly, DM, i.e. the most common cause of ESKD, and prior stroke were also exclusion criteria. In the overall trial, although the primary composite outcome of CV events as well as CV and total mortality were significantly lower in the intensive-treatment group compared with the standard-treatment group, kidney outcomes did not differ between the two groups. A sub-analysis of the SPRINT CKD subpopulation [26] showed no difference between groups in the primary outcome or in the pre-specified kidney outcome, but a lower total mortality rate in participants in the intensive BP arm. The above results must be interpreted with caution, since the SPRINT trial was not designed or powered to study kidney outcomes, and, as such resulted in an extremely small number of kidney events (15 vs 16 in the two groups).

Following the above, the 2023 ESH Guidelines indicate that a recommendation to target office SBP <120 mmHg in persons with CKD cannot be made. The reasoning for this is that the only relevant findings are delivered by this hypothesis-generating sub-analysis of the SPRINT trial, which included only a narrow range of the CKD population (non-diabetic, non-proteinuric CKD with eGFR 20–60 mL/min/1.73 m^2^) and had both the primary outcome and the main kidney outcome (with only few events) be not significantly different between treatment groups. Furthermore, although SPRINT according to protocol used a trial-specific automated BP measurement technique, during its execution no consistent universal methodology was followed with regards to personnel attendance (no attendance, attendance during rest periods, readings’ period or both), a fact that directly influenced the observed differences in outcomes [27]. It is also known that unattended SBP (assessed in about 42% of SPRINT participants) and conventional office SBP measurement can vary substantially in the individual (between 5 and 15 mmHg) [28].

With regards to persons with DM and CKD, the 2023 ESH Guidelines identify no direct evidence to answer the question of optimal target BP. Older studies, including the United Kingdom Prospective Diabetes Study (UKPDS) 38 [29] and the sub-analysis of participants with DM of the Hypertension Optimal Treatment (HOT) [30] trials offered insight on the DBP target, since they randomized in different on-treatment DBP levels. The Action to Control CardiOvascular Risk in Diabetes (ACCORD)-BP trial randomized high-risk patients with type 2 diabetes mellitus (T2DM) to target SBP <120 or <140 mmHg [31]. Apart from showing no difference in the primary outcome, most possibly due to interactions with other arms of the factorial design and the unexpectedly low event rate [32], ACCORD-BP excluded individuals with SCr >1.5 mg/dL; thus, it can offer very little insight into the optimal BP in patients with CKD and DM. A *post hoc* analysis of the Reduction of Endpoints in NIDDM with the Angiotensin II Antagonist Losartan (RENAAL) study showed that baseline SBP of 140–159 mmHg increased risk for ESKD or death by 38% compared with SBP <130 mmHg [33]. A relevant *post hoc* analysis from the Irbesartan Diabetic Nephropathy Trial (IDNT) showed that SBP >149 mmHg was associated with a 2.2-fold increase in the risk for doubling SCr or ESKD compared with SBP <134 mmHg; moreover, progressive lowering of SBP down to 120 mmHg improved kidney and patient survival, but below 120 mmHg, all-cause mortality increased [34]. Finally, although limited by the heterogeneity of the included studies [32], a recent meta-analysis in patients with CKD G3–G5 has reported a mortality benefit by a SBP reduction of 16 mmHg and an absolute SBP of <132 mmHg with a nonsignificant benefit at achieved SBP values of <125 mmHg [35]. In a more recent pooled analysis of four RCTs (AASK, ACCORD, MDRD and SPRINT), all-cause mortality showed a tendency to a reduction with intensive treatments (BP <130 mmHg), but this finding was not statistically significant. However, after excluding patients with higher GFR and those undergoing intensive glycemic control, lowering BP to <130 mmHg decreased all-cause mortality (hazard ratio 0.79, 95% confidence interval 0.63–1.00, *P *= .048) when compared with a standard target of <140 mmHg [36].

Taking these largely indirect findings together and considering that, at least after development of proteinuria, progression of kidney injury tends to follow the same course in different situations, the 2023 ESH Guidelines suggest that: (i) the BP target for proteinuric nondiabetic CKD applies to patients with proteinuric diabetic kidney disease as well and (ii) for both patient categories, a target SBP of <130 mmHg and DBP <80 mmHg, if well tolerated, can be associated with protection against CKD progression in individuals with an albuminuria >30 mg/g. A similar target may be associated with a reduction in mortality in most patients with CKD. Particularly in patients with advanced CKD (G4 and G5), careful monitoring of eGFR is recommended as a further functional, but reversible, decline of GFR may occur on a lower BP. Finally, most of the patients with CKD have CV comorbidities that require respective target BP values to be taken into account, and thus these CV comorbidities and not CKD protection may primarily guide the target BP in an individual patient.

However, the 2023 ESH Guidelines acknowledge that these recommendations have a number of limitations: (i) none of the trials comparing different BP targets included patients with diabetes and CKD, thus current evidence cannot be readily extrapolated to this subpopulation; (ii) MDRD and AASK trials randomized participants to different mean BP levels, which cannot be readily extrapolated to SBP and DBP values; (iii) MDRD and AASK trials recruited patient populations of a relatively young age (mean age 51.7 and 54.6 years, respectively), and thus, their findings cannot be readily extrapolated to older patients with CKD; and (iv) even for the long-term observational analyses, the benefits associated with lower BP targets were mainly apparent in individuals with proteinuria.

**Figure 2:**
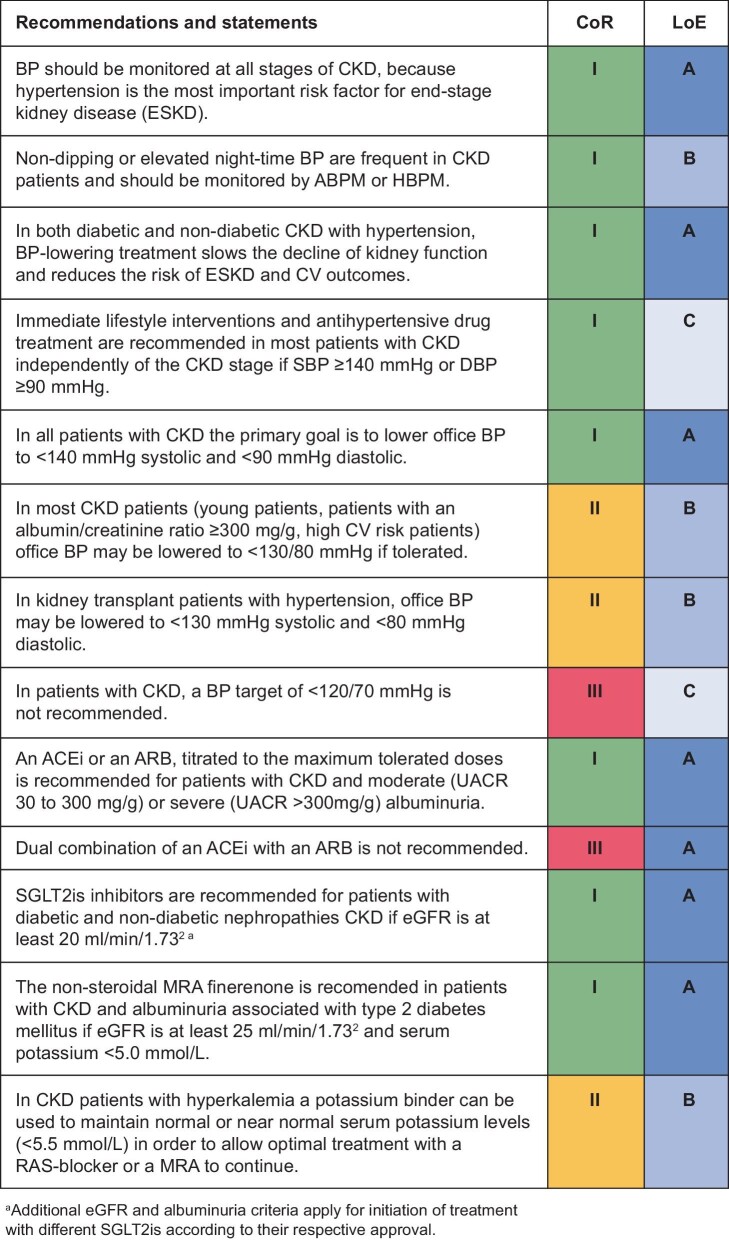
Treatment strategies in patients with hypertension and CKD (from [1], with permission).

Overall, as shown in Fig. 2, the guidelines recommend that in all patients with CKD the primary goal is to lower office SBP to <140 mmHg and DBP <90 mmHg (CoR I, LoE A) and that in most CKD patients (young patients, patients with a urine ACR ≥300 mg/g, high CV risk patients) office BP may be lowered to <130/80 mmHg if tolerated (CoR II, LoE B). Actively targeting an office SBP target of <120 mmHg and DBP <70 mmHg cannot be recommended because of the absence of relevant evidence and the potential to induce harm (CoR III, LoE C).

### Lifestyle interventions

The 2023 ESH Guidelines highlight a list of lifestyle interventions that are recommended in individuals with hypertension [1], including weight loss (CoR I; LoE A), a healthy dietary pattern (CoR I; LoE A), daily physical activity and structured exercise (CoR I; LoE B), reduction of alcohol intake close to abstinence (CoR I; LoE B) and smoking cessation (CoR I; LoE B). Dietary salt (NaCl) restriction to <5 g (∼2 g sodium) per day is recommended for all patients (CoR I; LoE B), and is emphasized for patients with CKD as it can be particularly helpful for BP control and reduction of albuminuria [37]. Increased potassium consumption, preferably via dietary modification, is recommended for adults with elevated BP, except for patients with advanced CKD (CoR I; LoE B).

### Antihypertensive agents

**Figure 3:**
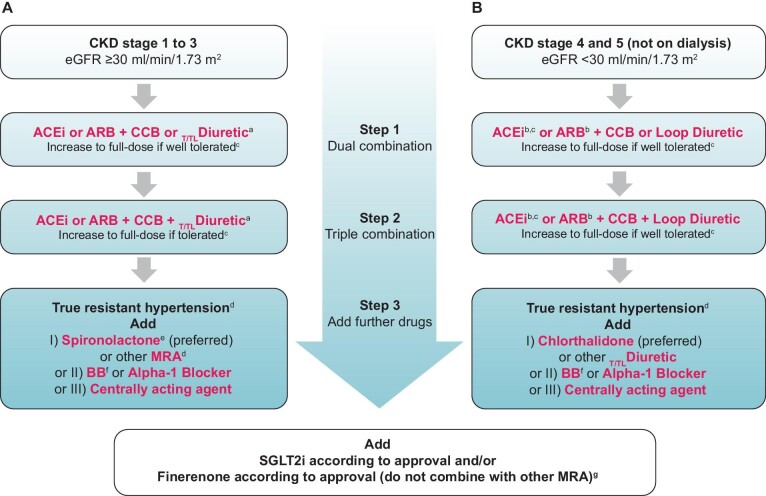
BP-lowering therapy in patients with hypertension and CKD. (**A**) Therapy for CKD G1–G3 (eGFR ≥30 mL/min/1.73 m^2^). (**B**) Therapy for CKD G4–G5 (eGFR <30 mL/min/1.73 m^2^) not on dialysis. (a) Transition from thiazide/thiazide-like (T/TL) diuretic to loop diuretic should be individualized in patients with eGFR <45 mL/min/1.73 m^2^. (b) Cautious start with low dose. (c) Check for dose adjustment according to renal impairment for drugs with relevant renal excretion rate. (d) When SBP is ≥140 mmHg or DBP is ≥90 mmHg provided that: maximum recommended and tolerated doses of a three-drug combination comprising a RAS blocker (either an ACEi or an ARB), a CCB and a T/TL diuretic were used, adequate BP control has been confirmed by ABPM or by HBPM if ABPM is not feasible, various causes of pseudo-resistant hypertension (especially poor medication adherence) and secondary hypertension have been excluded. (e) Caution if eGFR <45 mL/min/1.73 m^2^ or serum potassium >4.5 mmol/L. (f) Should be used at any step as guideline-directed medical therapy in respective indications or considered in several other conditions. (g) SGLT2is and finerenone should be used according to their approval for CKD treatment (from [1], with permission). T/TL: thiazide/thiazide-like.

The 2023 ESH Guidelines comment that existing evidence suggests that BP reduction with any type of first-line antihypertensive agents can offer similar protection in individuals with and without CKD against major CV events (stroke, myocardial infarction, heart failure or CV death) and all-cause death [38]. Achieving the recommended BP targets in CKD usually requires combination therapy, which should consist of a renin–angiotensin system (RAS) blocker with a calcium channel blocker (CCB) or a thiazide/thiazide-like diuretic, if eGFR levels are ≥45 mL/min/1.73 m^2^ (up to CKD G3a), while in patients with an eGFR <30 mL/min/1.73 m^2^ (CKD G4–G5), thiazide/thiazide-like diuretics should be generally replaced by loop diuretics, according to the updated algorithm presented in Fig. 3. The transition from treatment with a thiazide/thiazide-like to a loop diuretic should be individualized in patients with eGFR values between 30 and 45 mL/min/1.73 m^2^, with effectiveness in these and lower eGFR levels being established at least for chlorthalidone.

Following seminal clinical trials, in people with diabetic [39–42] and non-diabetic CKD [20, 43, 44] an angiotensin-converting enzyme inhibitor (ACEi) or an angiotensin-receptor blocker (ARB) is the treatment of choice, especially in those with moderately or severely increased albuminuria, where these agents were found to reduce proteinuria, the rate of GFR decline, and the risk of doubling of SCr or ESKD (CoR I; LoE A). The ACEi or ARB monotherapy should be at maximum tolerated approved doses to achieve optimal nephroprotection. Dual combination of an ACEi with an ARB or combination of aliskiren with any of the two is not recommended (CoR III; LoE A), because two relevant outcome trials were prematurely terminated as combination therapy was associated with increased risk of adverse events [45, 46]. In normoalbuminuric individuals with hypertension, ACEis or ARBs are able to delay the progression to severely increased albuminuria compared with placebo [47], but no evidence exists for better preservation of kidney function with RAS blockers compared with other major antihypertensive classes [48]. Currently, there is no evidence to stop treatment with RAS blockers in advanced CKD, as in a recent open-label trial in which patients with an eGFR <30 mL/min/1.73 m^2^ were randomized to either discontinuation or continuation of therapy with RAS inhibitors, discontinuation was not associated with a significant between-group difference in the long-term eGFR decline [49].

The 2023 ESH Guidelines highlight also the main therapeutic challenges with ACEi or ARB treatment [1]. As the vasodilating effect of ACEis or ARBs on the efferent arteriole reduces intraglomerular pressure, eGFR drops commonly by an average of 10%–15% in the first weeks of treatment with these agents (eGFR dip). A similar hemodynamic effect can be seen with large BP reductions offered by any antihypertensive agent. Thus, repeated monitoring of eGFR and blood electrolytes within 4–8 weeks (depending on baseline kidney function) is important when treatment is initiated. Clinicians should not be alarmed by this early GFR drop, but if the decline in GFR continues or is more severe (>30%), the RAS blocker should be stopped, and the patient should be investigated for the presence of renovascular disease. Use of RAS blockers in CKD patients further increases the risk of hyperkalemia [50]. Incident hyperkalemia is associated with increased mortality [51] and is the most frequent reason for dose reduction or discontinuation of ACEis and ARBs in CKD patients [52, 53]. However, reducing the dose or discontinuing RAS blockers has been associated with increased risk of CV events in large observational studies [53] and should be avoided. Novel potassium binders (patiromer and sodium zirconium cyclosilicate) were shown to effectively normalize elevated serum potassium and chronically maintain normal levels in CKD patients treated with ACEis, ARBs or spironolactone, with good tolerability [54, 55]. Thus, these agents can be used to maintain serum potassium <5.5 mmol/L in individuals with CKD [56, 57] (CoR II; LoE B).

The 2023 ESH Guidelines emphasize that most individuals with CKD will not achieve target BP control with ACEi or ARB monotherapy, and dual combination by adding a dihydropyridine CCB or a diuretic should almost always be used to initiate the treatment in hypertensive patients with CKD as in most patients with hypertension [58, 59]. Nevertheless, the majority of patients with CKD would need triple combination to achieve target BP (Fig. 3) [1]. Dihydropyridine CCBs were shown to increase proteinuria when used in the absence of a RAS blocker in patients with proteinuric CKD [41, 60]. However, in the general hypertensive population, where the majority of patients do not have moderately or severely increased albuminuria, dihydropyridine CCBs have similar effects on kidney outcomes with RAS blockers or diuretics [48]. Moreover, in a study of hypertensive patients of which 19% had moderately and 5% severely increased albuminuria at baseline, a combination of RAS blocker with a dihydropyridine CCBs was superior in reducing kidney outcomes compared with a RAS blocker–thiazide combination [61].

Diuretics are particularly useful in CKD patients, as these individuals are most often sodium-sensitive (especially if older, diabetic or obese) and have high prevalence of treatment resistant hypertension [59, 62]. Furthermore, diuretics were shown to effectively reduce proteinuria when added to RAS blockers in proteinuric CKD [37]. When GFR falls below 45 mL/min/1.73 m^2^, thiazide diuretics become less effective, as they cannot reach their tubular site of action due to competition for tubular secretion with other substances that accumulate in CKD [63]. This is theoretically also the case for thiazide-like diuretics, but recent RCT evidence indicated that at least chlorthalidone is clinically effective in lowering BP in patients with G4 CKD [64]. In general, in patients with CKD G3b (eGFR 30–44 mL/min/1.73 m^2^), diuretic therapy should be modified and the dosing individualized, while in patients with CKD G4 (eGFR <30 mL/min/1.73 m^2^), thiazides should be substituted with a loop diuretic. Within this class, torasemide might be preferred to furosemide because of its longer half-life, which allows a less frequent dosing scheme and a better adherence to treatment [65].

Finally, triple antihypertensive drug therapy may not control BP in a number of CKD patients. Hypertension is defined as resistant to treatment when appropriate lifestyle measures and treatment with optimal or best tolerated doses of three or more drugs (a thiazide/thiazide-like diuretic, a RAS blocker and a CCB) fail to lower office BP to <140/90 mmHg [1]. The inadequate BP control should be confirmed by uncontrolled 24-h BP (≥130/80 mmHg). Evidence of adherence to therapy and exclusion of secondary causes of hypertension are required to define true resistant hypertension. In patients with true resistant hypertension, the fourth line treatment should include the mineralocorticoid receptor antagonist (MRA) spironolactone, based on the evidence from the PATHWAY-2 (Spironolactone versus placebo, bisoprolol, and doxazosin to determine the optimal treatment for drug-resistant hypertension) trial [66] and relevant meta-analyses [67]. However, patients with an eGFR with eGFR <45 mL/min/1.73 m^2^ or potassium >4.5 mmol/L were excluded from this study [66] and, thus, the efficacy and safety of spironolactone in such individuals are not established. In the AMBER (Patiromer versus placebo to enable spironolactone use in patients with resistant hypertension and chronic kidney disease) trial that used spironolactone with addition of placebo or patiromer in patients with treatment resistant hypertension and eGFR 25 to ≤45 mL/min/1.73 m^2^, BP was effectively reduced in both groups, but the rates of hyperkalemia (potassium ≥5.5 mmol/L) were about 60% and 35%, respectively, at 12 weeks [68]. Based on the above, the 2023 ESH Guidelines have also updated the treatment algorithm for true resistant hypertension depending on underlying renal function. Use of spironolactone as a fourth antihypertensive agent in patients with CKD G3b and treatment-resistant hypertension is generally recommended only when necessary (when BP control is not achieved with addition of other agents) and should be done with caution and frequent potassium monitoring. Use of novel potassium binders is advisable to maintain serum potassium <5.5 mmol/L. Spironolactone is not recommended in patients with CKD G4 or higher. Instead, in the recent CLICK (Chlorthalidone in Chronic Kidney Disease) randomized trial that included 160 patients with CKD G4 and resistant hypertension, the addition of chlorthalidone (mean dose 23 mg daily) on top of previous antihypertensive treatment (including a loop diuretic) was associated with 10.5 mmHg reduction in 24-h SBP [64]; as such, the algorithm now suggests the addition of chlorthalidone for this group of patients [1].

Beta-blockers and alpha-blockers can offer important help towards BP lowering in patients with CKD, since sympathetic activity is commonly increased [69]; however, their effects in CKD have not been tested in trials with hard kidney outcomes. Bisoprolol (5–10 mg/day), doxazosin extended release (4–8 mg/day) or a centrally acting agent such as the alpha-adrenergic receptor agonists (clonidine 0.1–0.3 mg, or moxonidine 0.2–0.3 mg twice daily) can be used [70]. However, bisoprolol and doxazosin reduced BP less effectively than spironolactone in the PATHWAY-2 trial [66], while clonidine has shown similar BP-lowering effects to spironolactone in resistant hypertension, with several side effects [70]. Non-dihydropyridine CCBs (if used together with RAS blockers) were associated with reductions in proteinuria and decline of kidney function in proteinuric CKD [66, 67], but when added to a RAS blocker in normoalbuminuric hypertensive subjects do not seem to offer additional nephroprotection [71]. Direct vasodilators, such as hydralazine or minoxidil, should be used parsimoniously because they may cause severe fluid retention and reflex sympathetic activation with tachycardia. Recent RCTs have shown that endovascular renal denervation (RDN) can be associated with a significant, albeit not marked, office and ambulatory BP reduction in patients with uncontrolled hypertension [69, 72, 73]. In a large registry of renal denervated patients, the BP reduction was long-lasting and devoid of significant safety problems [71, 74]. RDN can thus be proposed as an adjunctive therapy to patients with resistant hypertension provided eGFR >40 mL/min/1.73 m^2^, in whom BP control cannot be achieved nor serious side effects cannot be avoided with antihypertensive medications [72, 75].

## USE OF ADDITIONAL DRUGS THAT OFFER NEPHROPROTECTION AND CARDIOPROTECTION IN CKD

In addition to BP control at the targets and with the agents described above, the 2023 ESH Guidelines included for the first time a considerably detailed discussion and highlighted that progression of CKD and risk of CV events and mortality can be reduced in CKD patients by two novel drug classes that also have some BP-lowering effects, although they are not approved as antihypertensive agents [1].

The 2023 ESH guidelines discuss that early clinical studies in patients with T2DM with the oral antihyperglycemic class of sodium-glucose co-transporter 2 inhibitors (SGLT2is), suggested that these agents can offer office BP reductions of around 3–5/1–2 mmHg [76], that were later confirmed with ABPM studies [77]. Of interest, larger reductions were described in patients with CKD G4 (around 7 mmHg for SBP) [78]. The main mechanism is a mild natriuretic/diuretic effect occurring possibly from both inhibition of proximal sodium reabsorption and osmotic diuresis [76]. These agents were also shown to reduce urine ACR by 25%–40%, depending on the baseline albuminuria levels [79], as well as plasma uric acid, which is also important in CKD patients [80]. The Guidelines refer to the fact that CV outcome trials with SGLT2is in patients with T2DM (which included also large proportions of patients with CKD), showed large and homogeneous reductions of around 40% in composite kidney endpoints [81–83]. The document also analyzes the results of the kidney outcome trials investigating SGLT2is on diabetic and non-diabetic CKD on top of standard therapy including an ACEi or an ARB on maximum tolerated doses, i.e., the CREDENCE (Canagliflozin and Renal Events in Diabetes with Established Nephropathy Clinical Evaluation) [84], DAPA-CKD (Dapagliflozin and Prevention of Adverse Outcomes in Chronic Kidney Disease) [85] and EMPA-KIDNEY (The Study of Heart and Kidney Protection With Empagliflozin) studies [86], to conclude that all three trials were prematurely terminated due to benefit and showed significant reductions compared with placebo on composite kidney outcomes and individual endpoints such as doubling of SCr and progression to ESKD. In the EMPA-KIDNEY trial, the reduction in the composite kidney outcome was evident in patients across the whole range of eGFR and most striking in patients with severely increased albuminuria. The chronic rate of eGFR loss was lower with empagliflozin in all urine ACR subgroups [86]. A mild eGFR drop may also be present during the first weeks of treatment, but managed as in the case of RAS blockers. The mild BP reduction is suggested as a contributor to the nephroprotective effect of SGLT2is. It is highlighted that in CREDENCE and DAPA-CKD, SGLT2is were also able to reduce the risk of some CV events and in DAPA-CKD the risk of mortality in patients with CKD [87], something that was not previously evident with RAS blockade or any other drug treatment in this population [88–91].

The Guidelines report that addition of a steroidal MRA (spironolactone or eplerenone) on top of an ACEi or an ARB in patients with proteinuric diabetic CKD showed significant reductions in urine albumin or protein excretion [92–94], independently of the BP-lowering effect, but their use was restricted in clinical practice due to absence of evidence from hard outcome trials and the increased risk of hyperkalemia [95]. The main mechanism of this action was inhibition of several deleterious genomic and non-genomic effects of aldosterone breakthrough, including kidney tissue inflammation and fibrosis mediated through MR overactivation [96]. Finerenone is a novel, non-steroidal MRA with different duration of action and tissue distribution from steroidal MRAs, which inhibits binding of different coregulatory molecules to MR receptors allowing reduction in inflammatory and fibrotic processes, with less interference with the classical MR-mediated actions in the distal tubule than steroidal MRAs [96, 97]. The BP reduction observed with finerenone appears to be less than with spironolactone and does not seem to substantially contribute to its organ-protective effects [98]. Following evidence showing dose-dependent reductions of albuminuria [99], the Guidelines discuss the effect of two RCTs, FIDELIO-DKD (Finerenone in Reducing Kidney Failure and Disease Progression in Diabetic Kidney Disease) and FIGARO-DKD (Finerenone in Reducing Cardiovascular Mortality and Morbidity in Diabetic Kidney Disease), that tested finerenone in T2DM patients with CKD and moderately or severely increased albuminuria on top of ACEi or ARB treatment. In the FIDELIO-DKD trial, finerenone was associated with significant reductions in the risk of the primary kidney outcome, as well as in the risk of the secondary composite CV outcome versus placebo [100]. The overall difference in BP over the course of the trial was 2.7/1.0 mmHg favoring finerenone, and these effects were consistent across all groups of baseline BP [98]. Hyperkalemia leading to discontinuation of the trial regimen was 2.3% with finerenone and 0.9% with placebo and no fatal hyperkalemia adverse events were reported [100]. In FIGARO-DKD, finerenone was associated with a 13% significant reduction in the risk of the primary CV outcome, with consistent beneficial effects on kidney outcomes and similar tolerability profile [101]. In the FIDELITY (FInerenone in chronic kiDney diseasE and type 2 diabetes: Combined FIDELIO-DKD and FIGARO-DKD Trial programme analYsis) on-treatment analysis combining the patient population of both trials, finerenone reduced mortality by 18% compared with placebo [102]. Other non-steroidal MRAs (esaxerenone and apararenone) have also been shown to significantly reduce albuminuria in CKD patients in phase 2 clinical trials [96], but have not yet been tested in hard kidney outcome studies.

In view of the above evidence, the 2023 ESH Guidelines recommended using SGLT2is or finerenone in patients with CKD in addition to lifestyle interventions and antihypertensive drug therapy. Use of an SGLT2i is recommended in patients with diabetic and in patients with nondiabetic CKD with a moderate or severe increase of albuminuria if eGFR is at least 20 mL/min/1.73 m^2^, with respect to current marketing authorizations of each agent (CoR I, LoE A), while use of finerenone is recommended in patients with CKD associated with T2DM and moderate or severe albuminuria, if eGFR is at least 25 mL/min/1.73 m^2^ and serum potassium is <5.0 mmol/L (CoR I, LoE A). The order of addition of an SGLT2i or finerenone has not been tested in clinical trials and can be based on the individual patient characteristics, including the need for improvement of glycemic control, potassium levels or persistent albuminuria.

## HYPERTENSION IN KIDNEY TRANSPLANT RECIPIENTS

The 2023 ESH Guidelines also discuss in considerable length the management of hypertension in kidney transplant recipients (KTRs) [1]. The guidelines discuss that kidney transplantation *per se* is associated with significant improvements in BP (8/5 mmHg in ambulatory BP) in the short- and mid-term post-transplant periods along with reduction in antihypertensive agents [103, 104]; as such, ambulatory BP in KTRs is significantly lower than that in carefully matched hemodialysis patients and similar to patients with CKD with matched kidney function [105, 106]. Despite these improvements, hypertension represents the most prevalent comorbidity post transplantation, with ABPM studies estimating hypertension prevalence in >95% of KTRs [107]. Elevated BP is associated with kidney function decline, target-organ damage, CV events and reduced graft and patient survival [108–110]. As such, hypertension may play an important role towards the significantly higher residual CV risk in KTRs than in general population [111].

The Guidelines report evidence on commonly encountered misclassification of hypertension status by office BP in KTRs [112], mostly due to a particularly high proportion of masked hypertension (20%–40%) [113]. This is associated with frequently impaired dipping status (around 50%) [113] and high rates of nocturnal hypertension (up to 70%–80%) [114, 115]. As ambulatory BP is a much stronger predictor of kidney function decline and target organ damage than office BP in KTRs [109], the guidelines advocated increasing the use of ABPM in KTRs for diagnosis and management of hypertension. With regards to the pathogenesis of hypertension in KTRs, the Guidelines highlight its multifactorial nature, involving traditional risk factors, factors related to CKD (most commonly, impaired sodium handling and activation of RAS and sympathetic nervous system and factors related to transplantation and its treatment [116]. Among major immunosuppressive classes, purine pathway inhibitors (mycophenolate mofetil or azathioprine), and mammalian target of rapamycin (mTOR) inhibitors (everolimus or sirolimus) do not affect BP control [116, 117]. The association of corticosteroid treatment with increased BP is emphasized, and partial activation of mineralocorticoid receptors by cortisol causing sodium retention is suggested as a main mechanism [110], while glucocorticoid avoidance or withdrawal protocols in KTRs are associated with better BP profile [118, 119]. Calcineurin inhibitors (cyclosporine or tacrolimus) are also associated with BP elevations, through increased sodium reabsorption via the thiazide-sensitive sodium chloride co-transporter in the distal convoluted tubule and upregulation of vasoconstrictive substances leading to increased total peripheral resistance and vasoconstriction of afferent arterioles [116, 117]. The effects of tacrolimus on BP appear less pronounced compared with cyclosporine.

As there are no specific RCTs that have tested different BP targets on major clinical endpoints in KTRs, BP targets for hypertension management in these individuals are extrapolated from data in CKD populations [1]. A target BP of <130/80 mmHg is considered as a reasonable target for KTRs (CoR II, LoE B). Lifestyle modifications should be adopted on the basis of recommendations for CKD, and combinations of major antihypertensive agents should be employed in most patients. The benefits of ACEis/ARBs in KTRs are still not clearly established, since observational and outcome studies provided conflicting results [110, 117]. In a recent meta-analysis of RCTs, the risk of graft loss was reduced by 38% with ACEi/ARBs, without any significant effects on non-fatal CV outcomes or death, whereas the incidence in hyperkalemia increased [120]. CCBs have consistently been associated with benefits such as improved graft survival and minimization of the preglomerular vasoconstrictive effects of calcineurin inhibitors, especially in the early transplantation period. In the aforementioned meta-analysis, CCBs reduced the risk for graft loss by 42%, while in head-to-head comparisons with ACEis/ARBs, CCBs significantly increased GFR by 11 mL/min [120]. Thiazide/thiazide-like diuretics are also effective and useful in patients with kidney transplantation, because they block the cyclosporine-mediated sodium retention. As no data are currently available on the effect of antihypertensive drugs on long-term kidney outcomes in KTRs, the guidelines avoid making any specific recommendation on preferred agents [1]. A notion is also made that transplant renal artery stenosis is not uncommon in KTRs and it should be effectively sought for in cases of uncontrolled or abrupt onset hypertension [116]; percutaneous renal artery angioplasty has high success rates in these patients [121].

## RENOVASCULAR DISEASE

The 2023 ESH Guidelines also discuss the prevalence [122, 123], prognosis [124] and management [125, 126] of the two main causes of renovascular hypertension, atherosclerotic renal vascular disease (ARVD) and fibromuscular dysplasia (FMD) [1]. Revascularization with balloon angioplasty without stenting is emphasized as the treatment of choice for patients with FMD and critical renal artery stenosis [127], while for ARVD, the recommendation is to offer revascularization on top of medical therapy in patients with documented secondary hypertension due to ARVD or those with high-risk clinical presentations (flash pulmonary edema, refractory hypertension or rapid loss of kidney function) with documented high-grade stenosis (≥70%) [1]. Medical therapy alone could be used for individuals with asymptomatic ARVD with stenosis <70%, patients with mild or moderate hypertension that is easily controlled with antihypertensive drugs and low-grade stenosis, or patients with non-viable kidney parenchyma, where revascularization has little to offer. In all the latter cases, if treatment initiation with an ACEi or an ARB results in eGFR reduction of ≥30%, careful re-evaluation is warranted. Current strategies in the management of ARVD are detailed in a recent clinical practice document by the European Renal Best Practice and the Working Group ‘Hypertension and the Kidney’ of ESH [128].

## CONCLUSIONS

The 2023 ESH Guidelines for the Management of Arterial Hypertension document includes important information and several recommendation updates regarding the management of hypertension in CKD, following the evolution of evidence in recent years (Table 2). Updated recommendations for daily nephrology practice that were briefly summarized in this text are relevant to the optimal BP targets, the algorithm of antihypertensive drug use in patients with CKD G3b and G4, the use of nephroprotective and cardioprotective agents such as SGLT2is and finerenone, management of hypertension in KTRs and current treatment of renovascular disease. Several other topics that are not discussed herein due to reasons of space can be also useful to practicing nephrologists, including management of hypertension in patient phenotypes that are commonly encountered (patients with diabetes, obesity, advanced CVD, sleep apnea), management of secondary hypertension, follow-up algorithms, the importance of adherence to treatment and the effects of the COVID-19 pandemic, among others. For all these topics, the reader is referred to the main Guideline document [1]. Implementation of the above recommendations in clinical practice is expected to help towards the improvement of BP control and reduction of hypertension-associated morbidity in patients with CKD.

**Table 2:** Important changes and additions in recommendations relevant to hypertension in CKD patients in the 2023 ESH Guidelines in relation to previous Guideline versions (for class or recommendation and level of evidence grading, if available, see Fig. 1 and text).

**Therapeutic area**	**Recommendation**
BP targets in CKD	In all patients with CKD the primary goal is to lower office BP to <140/90 mmHg
	In most patients with CKD (especially, young patients, patients with an ACR ≥300 mg/g, high CV risk patients) office BP should be lowered to <130/80 mmHg if tolerated
	In kidney transplant patients with hypertension, office BP should be lowered to <130/80 mmHg
	In patients with CKD, a BP target of <120/70 mmHg is not recommended
Antihypertensive drug use in CKD	Step 1 of treatment includes combination of an ACEi or ARB + CCB or _T/TL_Diuretic if eGFR ≥30 mL/min/1.73 m^2^, or combination of an ACEi or ARB + CCB or loop diuretic if eGFR <30 mL/min/1.73 m^2^^a^
	Step 2 of treatment includes combination of the 3 above drug classes to maximum tolerated doses
	Step 3 of treatment includes addition of spironolactone if eGFR ≥30 mL/min/1.73 m^2^ and potassium within the normal range or chlortalidone if eGFR <30 mL/min/1.73 m^2^^a^
Kidney and heart protection	SGLT2is are recommended for patients with diabetic and nondiabetic CKD, if eGFR is at least 20 mL/min/1.73 m^2^
	The non-steroidal MRA finerenone is recommended in patients with CKD and albuminuria associated with T2DM, if eGFR is at least 25 mL/min/1.73 m^2^ and serum potassium <5.0 mmol/L
Potassium management	In CKD patients with hyperkalemia a potassium binder can be used to maintain potassium <5.5 mmol/L to allow continuation of treatment with a RAS blocker or a MRA to continue
ARVD	Revascularization on top of medical therapy should be offered in patients with secondary hypertension due to ARVD or those with high-risk clinical phenotypes (flash pulmonary edema, refractory hypertension or rapid loss of kidney function) with documented high-grade stenosis (≥70%)
	Medical therapy alone could be used for individuals with asymptomatic ARVD with stenosis <70%, patients with mild/moderate hypertension, easily controlled with antihypertensive drugs and low-grade stenosis, or patients with non-viable kidney parenchyma

^a^Excludes patients with CKD G5 on dialysis.

_T/TL_Diuretic: thiazide or thiazide like diuretic.

## Supplementary Material

gfae041_Supplemental_File

## Data Availability

No new data were generated or analyzed in support of this research.
