# Supplementary material for: A European Renal Association (ERA) synopsis for nephrology practice of the 2023 European Society of Hypertension (ESH) Guidelines for the Management of Arterial Hypertension
Source: Nephrol Dial Transplant. 2024 Feb 14;39(6):929–43. doi: 10.1093/ndt/gfae041 (PMC11139525; doi:10.1093/ndt/gfae041)

**SUPPLEMENTARY APPENDIX**

**Supplementary Figure 1.** Evidence grade system with class of recommendation (CoR) and level of evidence (LoE) used in the 2023 ESH Guidelines. (from [1], with permission). BP, blood pressure, CVD, cardiovascular disease, HMOD, hypertension mediated organ damage, RCT, randomized controlled trial.

a) Limitations affecting the level of evidence include (but may not be limited to) high risk of bias, inability to account for important confounding factors in observational studies, questionable external validity and uncertain effect estimates (confidence intervals including negligible effect).


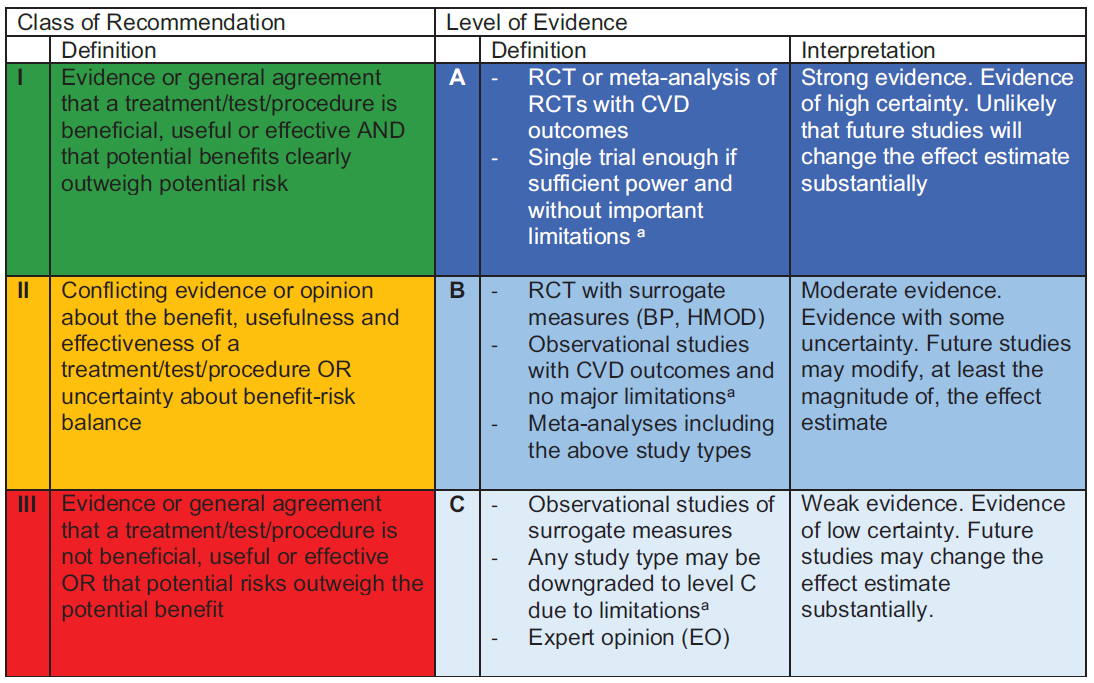

Supplement: gfae041_Supplemental_File [file gfae041_supplemental_file.docx]
